# COVID-19-induced multisystem inflammatory syndrome in a child with Wilson disease: a case report

**DOI:** 10.1186/s43066-022-00214-y

**Published:** 2022-09-09

**Authors:** Tawhida Yassin Abdel-Ghaffar, Haidy Mohammed Zakaria, Eman Mohamed Elsayed, Sondos Magdy, Suzan El Naghi, Suhaib Alsayed Mohammed Naeem, Mahmoud Yosry Hasan, Rabab Qasim khallaf

**Affiliations:** 1 Dr. Yassin Abdel Ghaffar Charity Center for Liver Disease and Research, 6 Emarat El Tasnieea St. from Makram Ebeid, Nasr City, Cairo 11566 Egypt; 2 Department of Pediatrics, Pediatric Hepatology, Faculty of Medicine, Ain Shams University, Cairo, 11591 Egypt; 3 Department of Clinical Research and Health Development, Menoufia Directorate of Health Affairs, Ministry of Health and Population, Shebin El-Kom, Menoufia 32511 Egypt; 4 Department of Pediatrics, Pediatric Cardiology, Faculty of Medicine, Ain Shams University, Cairo, 11591 Egypt; 5 Department of Pediatrics, Pediatric Intensive Care, Faculty of Medicine, Ain Shams University, Cairo, 11591 Egypt; 6 Department of Pediatrics, National Hepatology and Tropical Medicine Research Institute (NHTMRI), Cairo, Egypt; 7 Department of Histology, Faculty of Medicine, Al-Azhar University, Cairo, 71524 Egypt; 8 Department of Pediatrics, El-Shohda Central Hospital, Ministry of Health and Population, El-Shohda, Menoufia 32841 Egypt

**Keywords:** COVID-19, MIS-C, Multiorgan failure, Wilson’s disease

## Abstract

**Background:**

Infection with coronavirus disease 2019 (COVID-19) can progress to the multisystem inflammatory syndrome in children (MIS-C). Patients with liver cirrhosis are at increased risk of complications.

**Case presentation:**

We report on a 13-year-old Wilson’s disease patient who was referred for liver transplantation because of rapid deterioration in his hepatic condition. After admission, he developed fever, respiratory distress, coronary arteries dilatation on echocardiography, laboratory evidence of inflammation, and positive severe acute respiratory syndrome coronavirus (SARS-CoV-2) PCR. SARS-CoV-2-induced MIS-C was diagnosed. Inspite of aggressive management of MIS-C, progressive deterioration of the respiratory, liver, kidney, and cardiac functions occurred and he passed away.

**Conclusion:**

MIS-C is a serious possible complication leading to multiorgan failure and higher death rate especially in cirrhotic children. So, early diagnosis and management with higher level of care by a multidisciplinary team are warranted.

## Introduction

Novel severe acute respiratory syndrome coronavirus (SARS-CoV-2) is a newly identified disease characterized by symptoms of viral pneumonia, abnormal coagulation, and potential long-term damage to the heart, liver, and kidneys. Patients with well-controlled Wilson disease are not more susceptible to SARS-CoV-2 than the general population. However, patients with liver cirrhosis or severe comorbid disease are at increased risk of complications [1].

Infection with coronavirus disease 2019 (COVID-19) can progress to the multisystem inflammatory syndrome in children (MIS-C). The clinical presentation of MIS-C includes fever, severe illness, and the involvement of two or more organ systems, in combination with laboratory evidence of inflammation and epidemiologic or laboratory evidence of COVID-19 infection. Some features of MIS-C resemble Kawasaki disease, toxic shock syndrome, and secondary hemophagocytic lymphohistiocytosis/macrophage activation syndrome. Most MIS-C cases respond well to appropriate treatment if diagnosed early [2].

To date, the majority of MIS-C cases have been reported from North America and European countries with very few reports from African and Asian countries [3]. Here, we present the first case report of COVID-19-induced MIS-C in Egypt.

## Case presentation

A 13-year-old adolescent was diagnosed with a Wilson’s disease (WD) 4 months earlier after incidentally discovering elevated liver enzymes (laboratory results revealed low serum ceruloplasmin level, significant elevation of urinary copper before and after challenge with D-pencillamine and the histopathological findings in liver biopsy were suggestive of Wilson disease). He was started on D-pencillamine and zinc. Rapid decompensation of the liver occurred 3 months after beginning specific Wilson’s therapy with development of jaundice, ascites, and elevated international normalized ratio (INR) 1 month ago after the development of gastrointestinal (GIT) symptoms (abdominal pain and vomiting). At that point, he was referred to us for evaluation for liver transplantation (LT).

On examination, the patient was of average weight; 40 kg and height of 143 cm, fully conscious, normal vital signs, jaundiced, and ascetic. Both chest and heart examination showed no abnormality. There was no lower limb edema. He had a CHILD C score, and his MELD score was 39.

He was admitted to the ward. Chest computed tomography scan (CT) performed on admission (as routine investigation on admission in the era of COVID-19 infection) was normal. His laboratory parameters on admission are shown in Table 1.

**Table 1:** Laboratory parameters of the patient on admission and during follow-up

	On admission	Day 3	Week 1	Week 2	Week 4
**Hb (g%)**	7.4	8.3^a^	10.4	9	7. 4
**TLC × 10**^**3**^**(cells/cm)**	14.8	22.7	11.2	15.4	24
**Absolute neutrophils (×10**^**9**^**/L)**	11.84	19.3	7.1	11.8	
**Absolute lymphocytes (×10**^**9**^**/L)**	2.07	1.8	2.1	1.46	
**Platelets (×10**^**9**^**/L)**	233	311	159	102	53
**AST (IU/L)**	84		68	179	218
**ALT (IU/L)**	47		36	389	61
**ALKP (IU/L)**	145		191	159	
**GGT (IU/L)**	61			58	
**Albumin (g/dL)**	2.8	3.3^a^	2.5	2.6	2. 9
**Total bilirubin (mg/dl)**	27.5		18.3	40.6	57.6
**Direct bilirubin (mg/dl)**	10.8		8.8	18.2	25.8
**International normalized ratio**	5.5	3.1^a^	4.06	4.3	2. 6
**D-dimer (mg/L)**		0.71	2.84	2.25	
**Ferritin (ng/ml)**		233	248	350	
**C-reactive protein (mg/dl)**	54	55		16.2	31
**Sodium (mmol/L)**	115	117	134	134	125
**Potassium (mmol/L)**	3	3.4	4.2	4.2	3.1
**Calcium (mg/dl)**	7.1	9.6	8.3	8.7	8.8
**Phosphorus (mg/dl)**	3.6	3.4	2.8	1.9	5.5
**Magnesium (mg/dl)**	2.5	4.1	2.4	2.4	2.2
**Creatinine (mg/dl)**		0.6	0.5	0.6	2.5
**LDH (U/L)**		555	494	209	
**BUN (mg/dl)**		8	7	10	
**SARS-CoV-2 PCR**		Positive		Negative	Positive

*AST* aspartate transaminase, *ALT* alanine transaminase, *ALKP* alkaline phosphatase, *BUN* blood urea nitrogen, *GGT* gamma glutamyl transferase, *Hb* hemoglobin, *LDH* lactate dehydrogenase, *PCR* polymerase chain reaction, *SARS-CoV-2* severe acute respiratory syndrome coronavirus, *TLC* total leucocyte count

^a^After receiving blood and plasma

Thirty-six hours after admission, the patient was in poor general condition. Respiratory distress (RD) was noted (respiratory rate= 30–40 cycles/min) with intercostal retraction and diminished air entry on right side. He was fully conscious (GCS 15/15), tachycardic (heart rate=122 beats/min), and hypotensive (blood pressure 100/40 mmHg). He developed high temperature of 38°C, lower limb edema up to the knee, and tender hepatomegaly. Urgent chest X-ray, abdominal ultrasound, and echocardiography were requested.

Chest X-ray revealed a massive right side pleural effusion. Abdominal ultrasound showed cirrhotic liver, splenomegaly, calculous gallbladder, and moderate amount of ascites. At echocardiographic examination coronary arteries dilatation (left main coronary artery (*z* score +4.3) as well as right main coronary (*z* score +3.9)) was evident as well as minimal pericardial effusion and mild pulmonary hypertension.

**Fig. 1:**
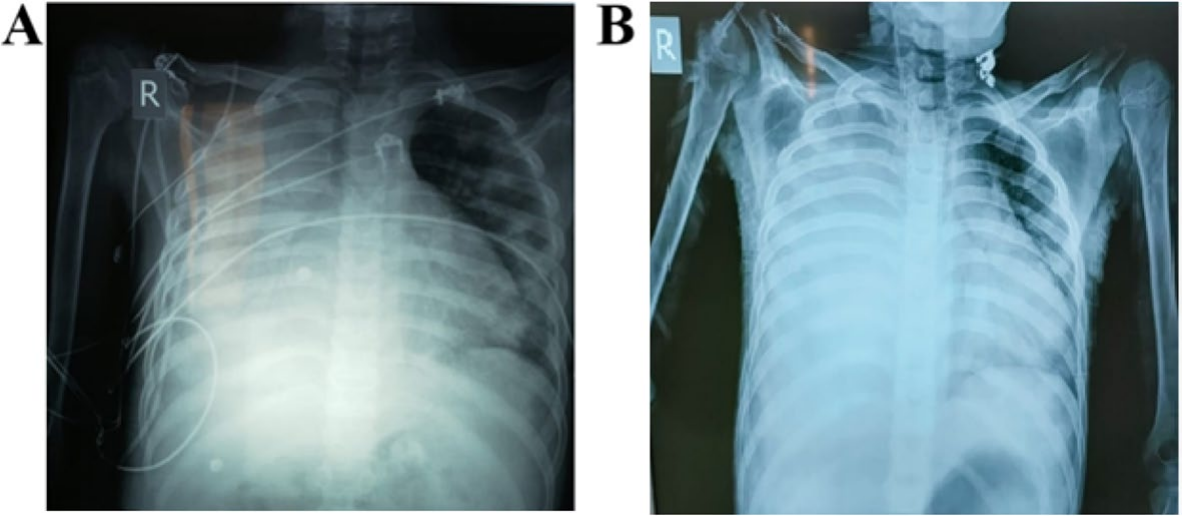
Chest X-ray of the patient showing massive right side pleural effusion. **A** Pleural effusion after the third day of pleurocentesis on the first presentation. **B** Reappearance of pleural effusion 4 weeks after first presentation

Urgent pigtail insertion in the right pleural space was done with drainage of 4 l of pleural fluid (Fig. 1A) and replacement with IV albumin 20% (5 cc/kg). The pleural fluid was transudate and free of organisms. Ascetic fluid (AF) aspiration was done. It was also transudate with SAAG = 2.1 denoting portal hypertension as its cause (Table 2).

**Table 2:** Analysis of aspirated ascetic and pleural fluid

	Ascetic fluid	Pleural fluid
**Color**	Deep yellow	Yellow
**Aspect**	Clear	Slightly turbid
**Cell count (cell/HPF)**	100	90
**Cell type**	Mainly lymphocytes	Mainly lymphocytes
**Gram stain**	no organisms	no organisms
**Glucose (mg/dl)**	142	130
**Protein (g/dl)**	0.8	0.6
**Lactate dehydrogenase (U/L)**	87	159
**Albumin (g/dl)**	0.7	0.9
**Fluid culture**	No growth	No growth
**Serum-ascites albumin gradient**	2.1	1.9

Serum albumin was 2.8 (g/dL) while testing the ascetic and pleural fluid samples

**Fig. 2:**
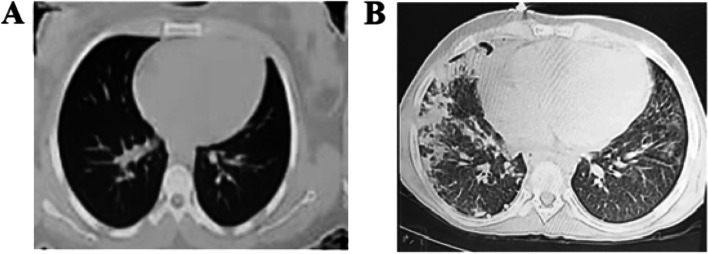
Chest CT of the patient. **A** At admission on the first presentation. **B** after 3 days of appearance of respiratory manifestations

Chest computed tomography scan done after pigtail insertion showed multiple patches of consolidation and ground glass opacities scattered in both lung fields more evident on the right side. It was suggestive of viral pneumonia mostly COVID-19 (COVID-19-Reporting and Data System (CO-RADS) V) (Fig. 2). A nasopharyngeal swab for SARS-CoV-2 was positive by reverse transcription-polymerase chain reaction (RT-PCR). Serological testing for COVID-19 immunoglobulin (Ig) M and IgG antibodies was also positive.

Laboratory parameters showed elevation of the inflammatory markers, anemia, electrolyte imbalance, elevated transaminases, hyperbilirubenemia, and coagulopathy (Table 1). Blood, stool, AF, and pleural fluid cultures were performed and showed no growth.

The patients had developed MIS-C as he had fever ≥ 38°C, severe illness necessitating hospitalization, cardiac (coronary arteries) and respiratory system affection, laboratory evidence of inflammation in the form of elevated CRP, D-dimer, ferritin, and LDH and negative cultures with positive SARS-CoV-2 test by RT-PCR.

The patient was then transferred to PICU in COVID isolation hospital, where he was aggressively managed according to the protocol for MIS-C (pulse steroid therapy (methylprednisolone 1000 mg/day for 3 days) followed by oral prednisone, intravenous immunoglobulin (2g/kg over 2 days), nasal oxygen, antibiotics (meropenem, clindamycin, and metronidazole), inotropes (milrinone 0.25 μg/kg/min), and vasoactive medications (noradrenaline 0.25 μg/ kg/ min) as he was hypotensive. He was also put on hepatic antifailure measures, octreotide infusion, and intravenous fluids with correction of the electrolyte imbalances.

Gradual improvement began to appear 2 days after the beginning of treatment. The patient became hemodynamically stable, and RD improved with adequate oxygen saturation. The amount of pleural fluid from drainage gradually decreased until it completely dried and then pig tail was removed. Hb level increased; TLC and transaminasis were decreasing, and electrolyte imbalance was improving but the INR, bilirubin level, creatinine, and serum ferritin were increasing gradually. He was weaned from oxygen and noradrenaline was stopped. Continuous improvement of the case had occurred till the second week of admission. After 2 negative COVID PCR tests, he was transferred to the ward.

In the ward while still on antibiotics and hepatic antifailure measures, he developed fever (38.5°C). Bilirubin and INR kept increasing. After a short period of improvement in creatinine and urine output, they both deteriorated. One week later, the patient developed tachycardia, hypotension, grade 1 hepatic encephalopathy, and bleeding from mouse and nose. Blood and plasma transfusion were received. Abdominal ultrasound showed mild to moderate ascites and grade II nephropathy. Echocardiography revealed mild pulmonary hypertension and still dilated both coronaries.

**Fig. 3:**
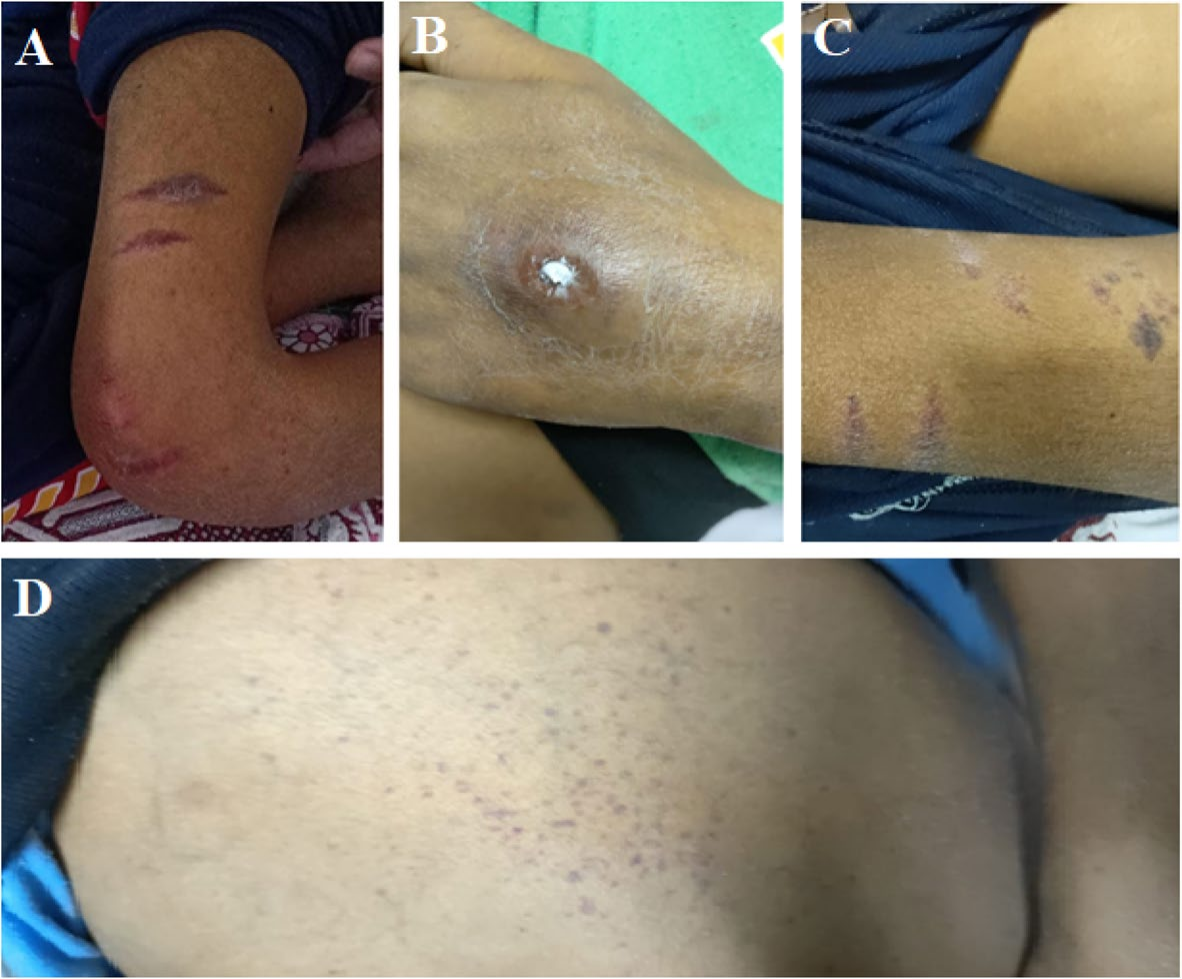
Skin lesions in the patient with MIS-C. Multiple skin lesions appeared in the form of petechiae (**D**) and ecchymotic patches (**A** &**C**) one of them shows ulceration (**B**)

**Fig. 4:**
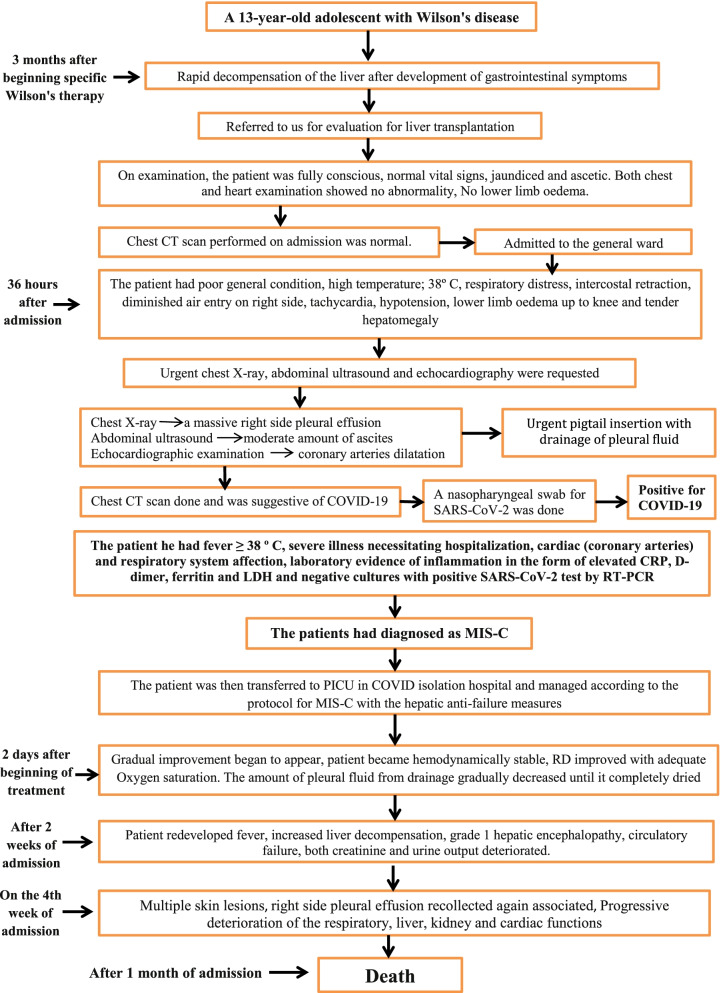
Flow chart showing the progression of the case

On the 4th week, multiple skin lesions appeared in the form of vasculitic rash, petechiae, and ecchymotic patches (one of them showed ulceration) (Fig. 3). Right side pleural effusion recollected again (Fig. 1B) associated with worsening hypoxia. Pleural drainage was done through intercostal chest tube, and respiratory support with oxygen was reinitiated. PCR for COVID-19 was repeated and showed positive results. The patient returned to isolation where progressive deterioration of the respiratory, liver, kidney, and cardiac functions occurred until he passed away after 1 month of admission (Fig. 4).

## Discussion

Patients with cirrhosis who acquire COVID-19 have poor outcomes. The clinical presentations of SARS-CoV-2 infection in cirrhotic patients slightly differ from that reported in the general population. Some authors reported absence of respiratory symptoms with normal chest CT on diagnosis. Others stated that fever was less frequent. An increase in cirrhosis complications rate was observed in most of cases [4].

Presence of fever ≥ 38 °C, severe illness necessitating hospitalization, cardiac and respiratory systems affection, and laboratory evidence of inflammation with positive SARS-CoV-2 test indicated the occurrence of COVID-19-induced MIS-C according to the Centers for Disease Control and Prevention criteria [5]. There are no specific criteria for differentiating MIS-C from acute deterioration of the chronic condition. However, cardiac and skin lesions favor MIS-C; they are not usual findings in acute deterioration of the liver disease.

The timing of onset of MIS-C symptoms relative to the acute SARS-CoV-2 infection is variable. In children who have a known history of documented or suspected COVID-19, the usual duration between acute infection and onset of MIS-C symptoms is 2 to 6 weeks and rare cases occurred after 6 weeks. This lag period coincides with the timing of acquired immunity and suggests that MIS-C may represent a post-infectious complication of the virus rather than acute infection [6].

In retrospect, we believe that our patient’s deterioration in his hepatic condition (for which he was referred to us) was induced by SARS-CoV-2 infection which presented by GIT and not respiratory symptoms. After a lag period and during his admission, he developed MIS-C.

SARS-CoV-2 results in a dysregulated immune response and higher expression of pro-inflammatory cytokines; interferon-γ, tumor necrosis factor (TNF), interleukin (IL)-1, IL-6, and IL-18 released into the blood stream. This cytokine storm syndrome causes multisystem inflammation and multiorgan failure [7].

It has even been suggested that radiological findings that may help distinguish between MIS from COVID-19 infections are pleural effusions, pericardial effusions, ascites, and hilar/mediastinal lymphadenopathies that favor MIS, reflecting an underlying multisystemic inflammatory process. The case series by Rostad et al. [8] described an incidence of pleural effusions in MIS-C children of 81.8% (the majority of the pleural effusions (67%) were bilateral) compared to 6.3% in COVID-19 infections (*P* < 0.01). The cytokine storm in COVID-19 patients can occur rapidly, with immune cells attacking the lungs soon after MIS-C [9].

Our patient had unilateral massive pleural effusion which could be either related to MIS-C or hepatic hydrothorax resulting from hepatic deterioration [10].

The cardiac affection in our case in form of pulmonary hypertension, pericardial effusion, decreased systolic ventricular function, and coronary arteries dilatation had progressed to circulatory failure that required ICU admission with inotropes and vasopressors support. The lower limb edema up to the knee was evident in the case. The uninhibited immune response to SARS-CoV-2 infection causes direct cardiotoxicity and rapid onset of severe cardiac dysfunction, hemodynamic instability, and vascular leakage with peripheral and pulmonary oedema [7].

The patient developed multiple skin lesions in the form of ecchymosis and necrotic lesions. Brumfiel et al. reported that morbilliform, pseudochilblain, urticarial, perniolike, vesicular, livedoid, papulosquamous, and necrotic lesions were described in large case series and systematic reviews [11].

Although the patient received adequate cardiac, respiratory, and fluid support, immunomodulatory therapy and broad-spectrum antibiotics besides the close monitoring and treatment adjustment by a multidisciplinary team, the condition progressed and severe deterioration of the liver functions occurred with development of renal failure. Both liver and renal affection may be due to a SARS-CoV-2 (a direct cytopathic effect on liver and kidney parenchyma through angiotensin-converting enzyme-2 protein receptors and/or microvascular injury caused by COVID-19-triggered cytokine storm, macrophage activation, and hypercoagulability) or the result of development of acute on chronic liver failure induced by COVID-19 infection [12].

MIS-C is more injurious in our cirrhotic patients as they have spontaneously increased proinflammatory response in comparison to non-cirrhotic patients due to the imbalance between proinflammatory and anti-inflammatory signaling pathways in immune cells. In addition, patients with Child-Pugh class C cirrhosis have activated monocytes which produce TNF-α [1].

The aggressive course of the condition and deterioration to death may be multifactorial as the original liver disease rapidly deteriorated possibly due to the superadded COVID-19 co-morbidity which may be in turn aborted the anti-inflammatory body response to the infection and consequently unopposed multi-organ failure.

## Conclusion

Cirrhotic children who develop COVID-19 have poor outcomes. MIS-C is a serious possible complication leading to sudden worsening of liver functions, multiorgan failure, and higher death rate. Increased index of suspicion and early diagnosis with higher level of care by a multidisciplinary team are warranted early.

## Data Availability

Not applicable.

## Abbreviations

AF: Ascetic fluid
CO-RADS: COVID-19-Reporting and Data System
COVID-19: Coronavirus disease 2019
CT: Computed tomography scan
GIT: Gastrointestinal
LT: Liver transplantation
IL: Interleukin
INR: International normalized ratio
MIS-C: Multisystem inflammatory syndrome in children
SARS-CoV-2: Severe acute respiratory syndrome coronavirus
RD: Respiratory distress
RT-PCR: Reverse transcription-polymerase chain reaction
TNF: Tumor necrosis factor
WD: Wilson’s disease
